# The interaction of chondroitin sulfate with a lipid monolayer observed by using nonlinear vibrational spectroscopy†

**DOI:** 10.1039/d1cp01975a

**Published:** 2021-06-07

**Authors:** Gergo Peter Szekeres, Szilvia Krekic, Rebecca L. Miller, Mark Mero, Kevin Pagel, Zsuzsanna Heiner

**Affiliations:** Institut für Chemie und Biochemie, Freie Universität Berlin Takustraße 3 14195 Berlin Germany kevin.pagel@fu-berlin.de; Department of Molecular Physics, Fritz-Haber-Institut der Max-Planck-Gesellschaft Faradayweg 4-6 14195 Berlin Germany; School of Analytical Sciences Adlershof, Humboldt-Universität zu Berlin Albert-Einstein-Straße 5-11 12489 Berlin Germany heinerzs@hu-berlin.de; Institute of Biophysics, Biological Research Centre Temesvári krt. 62 6726 Szeged Hungary; Doctoral School of Multidisciplinary Medical Sciences, University of Szeged Dugonics tér 13 6720 Szeged Hungary; Copenhagen Center for Glycomics, Department of Cellular and Molecular Medicine, Faculty Sciences, University of Copenhagen Blegdamsvej 3 DK-2200 Copenhagen N Denmark; Max Born Institute for Nonlinear Optics and Short Pulse Spectroscopy Max-Born-Straße 2a 12489 Berlin Germany

## Abstract

The first vibrational sum-frequency generation (VSFG) spectra of chondroitin sulfate (CS) interacting with dipalmitoyl phosphatidylcholine (DPPC) at air–liquid interface are reported here, collected at a laser repetition rate of 100 kHz. By studying the VSFG spectra in the regions of 1050–1450 cm^−1^, 2750–3180 cm^−1^, and 3200–3825 cm^−1^, it was concluded that in the presence of Ca^2+^ ions, the head groups together with the head-group-bound water molecules in the DPPC monolayer are strongly influenced by the interaction with CS, while the organization of the phospholipid tails remains mostly unchanged. The interactions were observed at a CS concentration below 200 nM, which exemplifies the potential of VSFG in studying biomolecular interactions at low physiological concentrations. The VSFG spectra recorded in the O–H stretching region at chiral polarization combination imply that CS molecules are organized into ordered macromolecular superstructures with a chiral secondary structure.

## Abstract 2

Chondroitin sulfate interacts with the headgroups of a lipid monolayer at the air–liquid interface and shows a chiral secondary structure.

## Graphical abstract

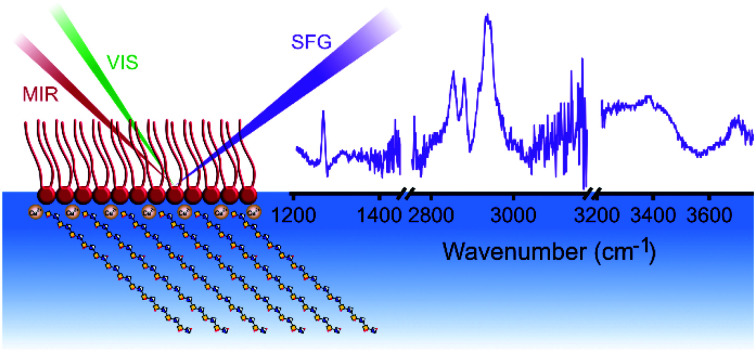

## Introduction

Glycosaminoglycans (GAGs) are linear, negatively charged polysaccharides, whose analysis and, therefore, understanding are greatly challenged by their high heterogeneity in chain length, degree of sulfation, and the resulting sulfation pattern. At the same time, their physiological importance and pharmaceutical relevance as anticoagulant or virus inhibitor clearly require more in-depth knowledge on their physico-chemical properties and *in vivo* behavior.

Most GAGs are in close proximity to the cell membrane either as a covalently bound ligand in a membrane- or extracellular-matrix-related proteoglycan, or as free molecules in the extracellular matrix. Although GAGs form a ∼50–500 nm thick network with proteins and lipids in the endothelial surface layer,^1^ they are usually not part of cellular membrane models. Therefore, there is an urgent need to understand the interactions of GAGs with other membrane components, *e.g.*, lipids, proteins, and the interfacial water molecules. This interfacial water structure provides insight into the electrostatic interactions and the resulting (de)hydration of the microenvironment.^2–5^ Vibrational spectroscopy is one of the best analytical tools to study the chemical composition and structure in a label-free way. Due to the high abundance of hydroxyl, sulfate, and carboxylate groups in GAGs, interface-sensitive vibrational spectroscopic studies of their interactions in the O–H stretching region are of particular importance. Even though some aspects of the interaction of GAGs with lipids were reported many decades ago,^6,7^ the high heterogeneity of GAG samples and the less advanced analytical instrumentation have rendered this research direction stagnating for the past few decades. More recently, however, the topic has gained renewed interest due to the presumed role of GAGs in atherosclerosis.^7–11^ Specifically, when the endothelium is damaged, proteoglycans and GAGs can migrate into the arterial intima, where they can interact with lipoproteins *via* a bridge formed by a divalent cation such as Ca^2+^.^10^ The complex can associate further with other lipophilic molecules, *e.g.*, cholesterol, and eventually grow to a size where its protrusion into the arterial lumen leads to insufficient blood flow. In addition, there has recently been enormous progress in techniques to produce homogeneous GAG chains for detailed structural studies.^12^ An increasing number of studies on lipid–GAG interactions and with that a considerable gain of knowledge is therefore expected in the next years.

Vibrational sum-frequency generation (VSFG) spectroscopy is a second-order nonlinear, label-free optical method for determining interfacial chemical structure, composition, and dynamics of molecules *in situ*. At interfaces, where centrosymmetry is broken, the detection and chemical analysis of molecular monolayers become possible even at very low surface coverages. During the past decade, secondary structures of peptides and proteins were increasingly investigated by chiral VSFG spectroscopy demonstrating the potential of this technique in revealing macromolecular structures and orientations at biological interfaces at the fundamental level *in situ* and in real time.^13^ Recent progress in laser technology made it possible to increase the laser repetition rate by two orders of magnitude to 100 kHz,^14,15^ which in turn led to a drastic increase in signal-to-noise ratio and a shorter acquisition time (≤10 s) in VSFG spectroscopic studies of solid-supported phospholipid monolayers.^16,17^

Here, for the first time, we applied high-repetition-rate chiral and achiral VSFG spectroscopy at the air–liquid interface to investigate how chondroitin sulfate (CS) interacts with a zwitterionic phospholipid monolayer made of dipalmitoyl phosphatidylcholine (DPPC). We investigated how the orientation of the head- and the tail-groups of phospholipids and the structure of interfacial water changed during the interaction with CS in the presence of Ca^2+^ ions. The VSFG spectra were collected in the 2750–3825 cm^−1^ and the 1025–1450 cm^−1^ ranges covering C–H and O–H stretching regions and characteristic bands in the fingerprint region, respectively, to provide complementary structural information. The spectra were acquired at 2.8 mM Ca^2+^ concentration and a CS concentration below 200 nM, which highlights the potential of VSFG spectroscopy in studying interfacial biomolecular interactions at low, physiologically relevant concentrations.

## Materials and methods

### Materials

In all experiments, Milli-Q water (18.2 MΩ cm) was used, and it is further referred to as “water”. Chloroform (spectroscopic grade, >99%), methanol (spectroscopic grade, >99.9%), anhydrous CaCl_2_, and chondroitin sulfate from shark cartilage (CS) were purchased from Sigma. Dipalmitoyl phosphatidylcholine (DPPC) was purchased from Avanti. Every chemical was used without further purification.

### Sample preparation for VSFG measurements

For the monolayer formation, first, 1 mg of DPPC was dissolved in 1 mL of the 1 : 1 mixture of chloroform and methanol. A Petri dish with a diameter of 35.4 mm was filled with 7 mL water, and 0.2 mL 0.1 M aqueous CaCl_2_ solution was added, resulting in a Ca^2+^ concentration of 2.8 mM, which corresponds to approximately twice the concentration found in the extracellular matrix.^18^ The reason we chose this concentration was to remain close to the physiologically relevant concentrations but also to be able to observe clearly which effects come from the DPPC–Ca^2+^ interaction and which from the DPPC–Ca^2+^–CS interactions. The combined volume filled the Petri dish completely without exhibiting large surface curvature. The amount of DPPC solution needed to reach the desired 30–32 mN m^−1^ surface pressure was determined by using a Langmuir–Blodgett instrument (Kibron MicroTrough XS, Helsinki, Finland). Prior to experiments in the ranges of 1050–1450 cm^−1^ and 2750–3200 cm^−1^, samples were left for at least 30 min for the organic solvents to evaporate and the DPPC monolayer structure to reach equilibrium before adding the CS solution to each sample. The equilibrium was considered set when two spectra collected 5 min apart yielded the same band shapes and intensities. In the experiments in the 3200–3825 cm^−1^ spectral range, the DPPC monolayer was formed on top of water, and then, after equilibrium was reached and VSFG spectra were collected, 200 μL 0.1 M Ca^2+^ solution was added to the sub-phase to reach the final 2.8 mM Ca^2+^ concentration. Then, when the monolayer reached equilibrium, 20 μL CS solution was added.

### Vibrational sum-frequency generation (VSFG) experiments

Vibrational sum-frequency generation spectroscopy is a nonlinear optical technique, where two laser pulses interact at the sample to generate a sum-frequency signal through the second-order nonlinear optical susceptibility, *χ*^(2)^, of the sample. As *χ*^(2)^ is nonzero only in non-centrosymmetric media in the dipole approximation, VSFG spectroscopy is interface sensitive. In a broadband VSFG measurement, femtosecond, spectrally broad mid-infrared (MIR) laser pulses, resonant with the vibrational mode(s) of the investigated molecule(s) overlap at the interface spatially and temporally with quasi-monochromatic visible or near-infrared (traditionally denoted as VIS) laser pulses. The molecular vibrational spectrum as a fingerprint, generated by the MIR pulses, is up-converted with the assistance of the VIS laser pulses to the visible spectral range *via* the sum-frequency process, where high-performance silicon-based detectors are available. VSFG measurements are performed using linearly polarized MIR and VIS pulses either in *p*- or *s*-polarization. The generated VSFG signal is detected either in *p*- or *s*-polarization. The corresponding polarization combination is denoted using a triplet of letters, where the first, second, and third letter corresponds to the polarization plane of the SFG, VIS, and MIR beams, respectively (*e.g.*, *ssp*). Performing the experiment using different laser polarizations enables the isolation of different linear combinations of the second-order susceptibility tensor elements, which in turn can be used to quantify molecular orientation and order. The resulting effective *χ*^(2)^ can also reveal the achiral (*ssp* and *ppp* in our measurements) and chiral character of the molecular layer. The chirality of the interface can be probed using *psp*, *spp*, and *pps* polarization combination. From the as-obtained VSFG spectrum, the vibrational resonance frequencies, amplitudes, and spectral widths can be determined, enabling the study of interfacial molecular structure and orientation.

Details of the operation of the high-repetition-rate VSFG spectrometer in the C–H– and O–H stretching and in the fingerprint regions were described elsewhere.^14,15^ Therefore, only a brief explanation is given here. The whole spectrometer is driven by a commercial diode-pumped Yb laser system operating at a centre wavelength of 1028 nm and a repetition rate of 100 kHz. The available 6 W of total output power was split in two parts. One part was used to generate quasi-monochromatic pulses around 514 nm using a home-built spectral compressor.^14^ The other part of the pump beam was applied to produce ultrashort, tuneable mid-infrared laser pulses either in the 2725–3825 cm^−1^ spectral range^14^ or in the 1000–1450 cm^−1^ spectral range *via* optical parametric amplification.^15^ To eliminate absorption of the infrared laser beam by atmospheric water vapor, a home-built purging-enclosure system was used along the mid-infrared beamlines. The mid-infrared pulse energies incident on the target were 0.2, 0.7, and 0.7 μJ at the centre wavenumbers of 1267, 2980, and 3455 cm^−1^, respectively, while the pulse energy of the visible pulses was kept at a constant value of 5 μJ. The visible and mid-infrared pulses were focused with an *f* = 300 mm and an *f* = 50 mm singlet lens, respectively, and then temporally and spatially overlapped at the surface of the sample. The angles of incidence of the visible and mid-infrared beams were 68° and 57°, respectively. The generated sum-frequency signal was imaged onto the slit of a spectrograph equipped with a Peltier-cooled, deep-depletion charge-coupled device (Horiba, Ltd). Zero-order half-waveplates were used in the incident beams to control their polarization planes. In addition, a polarizer and a half-waveplate were employed at the entrance of the spectrometer to select the appropriate polarization component of the sum-frequency beam, and to adjust the polarization of the sum-frequency beam transmitted through the polarizer for optimum diffraction efficiency at the spectrometer grating. The VSFG spectra were collected in *ssp* and *ppp* polarization combinations (corresponding to SFG, visible, and mid-infrared, respectively) in the entire spectral range, and an additional chiral polarization combination, namely *spp*, was applied in the O–H stretching region. The VSFG spectra were collected at acquisition times of 40 s, 10 s, and 20/30 s in the spectral region of 1025–1450 cm^−1^, 2750–3200 cm^−1^, and 3200–3825 cm^−1^, respectively. The spectral resolution of the spectrometer was ∼3 cm^−1^ limited by the spectral width of the visible pulses and the resolution of the spectrograph. All measurements were repeated several times in each spectral region at different sample positions to account for possible small variations in the environmental parameters (*e.g.*, room temperature) and to ensure the absence of sample inhomogeneity. All measurements were conducted at room temperature (23 °C) and a relative humidity of around 40%. All VSFG spectra shown in our study were first frequency calibrated based on a 50-μm-thick polystyrene film inserted in the infrared beam. Thereafter, the difference spectrum was calculated by subtracting the background, *i.e.*, without MIR excitation, from the raw VSFG spectrum. To produce comparable spectra in the different spectral regions, each difference spectrum was first divided by the acquisition time to convert the intensities into counts per second, while the non-resonant spectrum obtained at a silver surface was normalized to one and corrected with the actual infrared power measured at the sample. The difference VSFG spectra from the given spectral region were averaged and then normalized by the non-resonant spectrum measured on a silver surface.

## Results and discussion

**Fig. 1:**
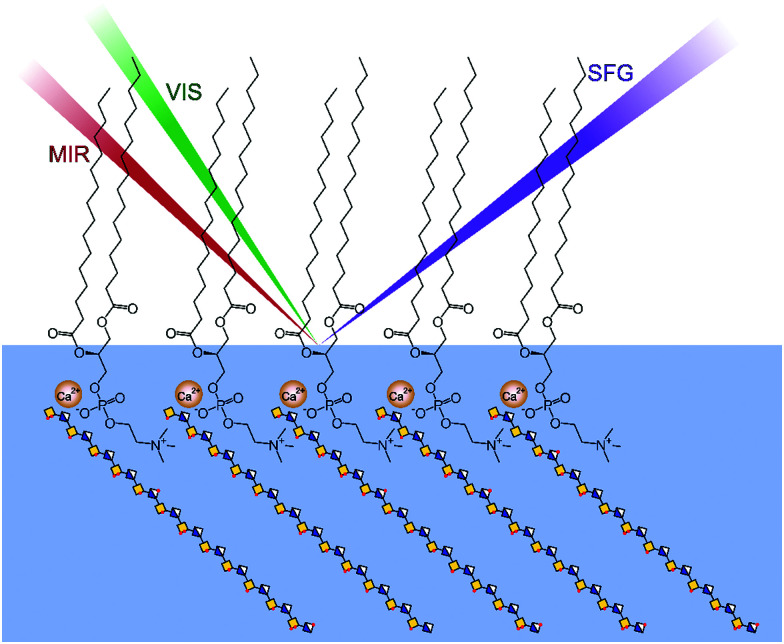
Schematic representation of the VSFG experiments on DPPC monolayers interacting with Ca^2+^ ions and chondroitin sulfate (CS) at the air–liquid interface.

In the experiments, the surface pressure of the DPPC monolayer, serving as a simple membrane model, was chosen to be ∼30 mN m^−1^ (ESI,† Fig. S1) to stay close to the surface pressure measured in cell membranes.^19^ The schematic representation of the experiments is shown in Fig. 1.

**Fig. 2:**
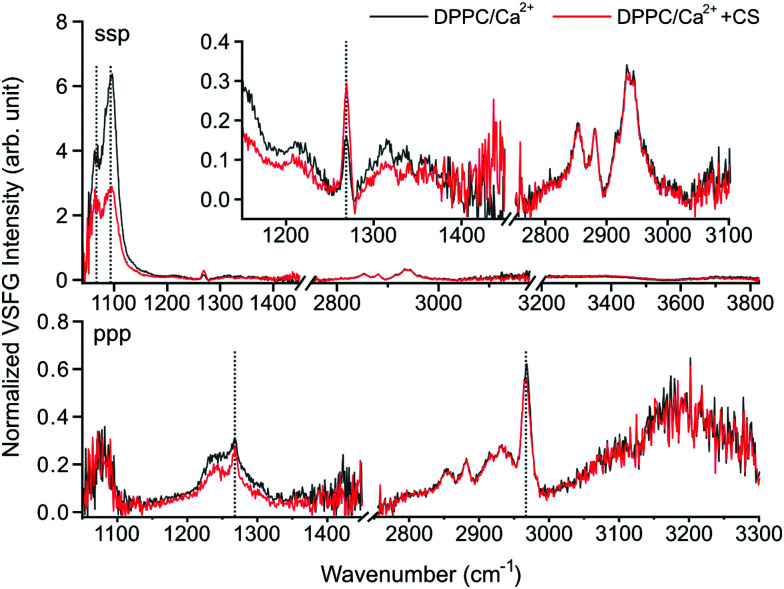
The *ssp* (top) and *ppp* (bottom) polarization VSFG spectra of a ∼30 mN m^−1^ DPPC monolayer over a 2.8 mM Ca^2+^ solution as subphase with (red trace) and without (black trace) CS. The inset shows the magnified sections of 1150–1450 cm^−1^ and 2750–3180 cm^−1^.

In Fig. 2, the combined VSFG spectra are shown of a ∼30 mN m^−1^ DPPC monolayer with 2.8 mM Ca^2+^ in the sub-phase in *ssp* polarization combination (*s* for sum-frequency, *s* for visible and *p* for mid-infrared) with and without CS in the 1025–1450 cm^−1^, 2750–3180 cm^−1^, and 3200–3825 cm^−1^ spectral ranges, and the corresponding *ppp* spectra in the 1050–1450 cm^−1^ and the 2750–3300 cm^−1^ ranges. The spectra cover most of the fingerprint, C–H stretching, and O–H stretching spectral regions in both polarization combinations, providing comprehensive information about the interfacial molecular groups corresponding to water, DPPC, and CS. The exact molar concentration of CS is difficult to determine due to its naturally high heterogeneity, but the 7.2 mg L^−1^ final concentration corresponds to a molar concentration below 200 nM.^20^

**Fig. 3:**
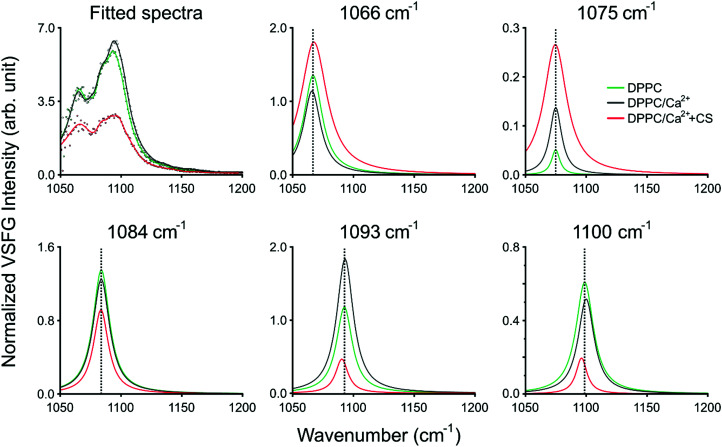
Results of the Lorentzian fitting of the VSFG spectra. The sum of all fitted peaks is shown on the top left. The different panels present the 1066 cm^−1^ (top middle), 1075 cm^−1^ (top right), 1084 cm^−1^ (bottom left), 1093 cm^−1^ (bottom middle), and 1100 cm^−1^ (bottom right) peaks fitted to the spectrum of DPPC on a 2.8 mM Ca^2+^ subphase without (black traces) and with (red trace) CS. As a control, the panels also contain the corresponding fitted spectrum and peaks of DPPC on pure H_2_O (green traces). The fitting parameters are listed in Table S1 in the ESI.†

In *ssp* polarization, the relative intensities of the 1066 cm^−1^, 1093 cm^−1^, and 1100 cm^−1^ vibrational bands strongly change upon the addition of CS, corresponding to the C–O–P in-phase stretching,^3^ the R–O–P–O–R symmetric stretching,^3^ and the PO_2_^−^ symmetric stretching modes,^3,4^ respectively. To better represent the spectral changes, multiple Lorentzian peaks were fitted to the spectra of DPPC on Ca^2+^ subphase with and without CS, and to the spectrum of DPPC on pure H_2_O subphase as control (fitting details are provided in ESI†). The fitting results are summarized in Fig. 3 and ESI,† Table S1. The 1066 cm^−1^ band shifts towards higher wavenumbers and, together with the 1075 cm^−1^ band, shows significant broadening due to additional vibrational modes from CS, *e.g.*, OSO_3_^−^ (a)symmetric stretching or C–O–S stretching^21^ (Fig. 3 top middle and right, ESI,† Table S1). In the meantime, the 1093 and 1100 cm^−1^ bands red-shift when CS is added (Fig. 3 bottom middle and right, ESI,† Table S1); since the phosphate group is highly sensitive to hydration, this shift indicates the contribution of the hydrogen bonding environment, which aligns around the phosphate group when the head group interacts with the negatively charged groups of CS. Simultaneously, the overall intensity of the spectrum between 1050 and 1100 cm^−1^ decreases due to the influence of CS. The vibrational bands at 1075 cm^−1^ and 1084 cm^−1^ do not show frequency shifts during the interaction of DPPC with Ca^2+^ and CS, and we assigned those to the symmetric stretching of the CO–O–C moiety^22^ and to C–O stretching.^23^ The same bands in *ppp* polarization remain unchanged (1066 cm^−1^ and 1093 cm^−1^), while the amplitude of the PO_2_^−^ symmetric stretching band is diminished. An additional vibrational mode with negative amplitude at 1112 cm^−1^ was discovered as a result of Lorentzian fitting and it is also visible in the obtained VSFG spectrum (*cf.* ESI,† Fig. S2), which was assigned to the OSO_3_^−^ asymmetric stretching, and to COH and CH deformations^21^ (see ESI,† Table S1). The negative amplitude is the result of an interference effect, which causes phase shift between the obtained vibrational bands of DPPC and CS.

A broad vibrational band is clearly visible around 1245 cm^−1^, which has negative and positive contribution in *ssp* and *ppp* polarization combination, respectively. We assigned it to PO_2_^−^ asymmetric stretching. The negative contribution comes from the interference between the CH_2_ deformation modes (above 1200 cm^−1^) in air and the PO_2_^−^ asymmetric stretch in water, where the phase jump at the air–liquid interface causes destructive interference. This effect vanishes when the VSFG spectra are collected in *ppp* polarization combination, since CH_2_ deformations in this plane are invisible (*i.e.*, they have zero projection in a plane parallel to the surface normal). Due to interference between the overlapping vibrational bands from the DPPC headgroups and the sulfate groups of CS, specifically those assigned to the OSO_3_^−^ antisymmetric stretching, C–H, and C–O–H deformations of GAGs,^21^ the negative amplitude at 1245 cm^−1^ is more enhanced and slightly blue-shifted when CS accumulates at the interface (*cf.*Fig. 2, and ESI† Fig. S2). Based on the expected orientation of the head groups at the interface in the presence of Ca^2+^ ions,^3^ and that the *ssp*/*ppp* intensity ratios of the 1066 cm^−1^ and the 1100 cm^−1^ vibrational modes decrease, we conclude that the angle between the surface normal and the head group (defined as P → N vector) increases due to the interaction with CS.

A sharp vibrational band can be observed at 1268 cm^−1^ which we assigned to the P=O stretching band.^24^ This peak does not show shifting in its vibrational frequency, but is strongly affected when the phosphate headgroup interacts with Ca^2+^ ions and also with CS (ESI,† Fig. S2), and these appear to have an opposite effect on its intensity compared to that of the 1066, 1093, and 1100 cm^−1^ bands. The orientation of the P=O bond is close to 45° relative to the surface normal, and this angle increases when CS is added. New bands and band shifts can be further observed at 1199, 1206, 1255, 1323, 1341, and 1366 cm^−1^. The majority of these bands come from different CH_2_ deformations due to *gauche* defects along the lipid tails.^25,26^ It is worth noting that without CS, the bands above 1300 cm^−1^ follow a repeating, somewhat periodic arrangement, while the addition of CS visibly changes the symmetric band profiles due to the C–H vibrational contribution from CS (Fig. 2 top).

Our hypothesis on the vibrational modes of the phosphate group is also confirmed by the VSFG data obtained at the pure DPPC monolayer system in comparison with the DPPC–Ca^2+^ and the DPPC–Ca^2+^–CS systems. (ESI,† Fig. S2). These observations exclude the possibility that the CS molecules introduce high level of disorder in the DPPC monolayer and suggest the reorientation of the headgroups instead. The bands in the spectral region of 1200–1400 cm^−1^ have so far not been observed for phospholipid monolayers at the air–liquid interface using VSFG spectroscopy and were only visible here due to the high laser repetition rate and signal-to-noise ratio.

The spectra in the C–H stretching region (Fig. 2 top, here shown between 2750–3180 cm^−1^), such as the 2850 cm^−1^ and 2920 cm^−1^ CH_2_ symmetric and asymmetric stretching modes, or the 2870 cm^−1^ symmetric CH_3_ stretching mode remain mostly unchanged.^27^ However, a slight decrease was observed in the intensity of the band at 2968 cm^−1^ assigned to the asymmetric CH_3_ stretching mode deriving from the lipid tails.^17^ From the intensity ratios between the CH_2_ and CH_3_ symmetric stretching bands^17^ we can conclude that the lipid monolayer is mostly in all-*trans* conformation with relatively small number of *gauche* defects, and remains almost unchanged during the interaction with CS. This is also confirmed by our results based on the CH_2_ deformation modes (above 1300 cm^−1^). Given that at ∼30 mN m^−1^ surface pressure the DPPC forms a continuous film on top of the sub-phase,^28^ it is expected that the reorientation of the lipid tails is sterically somewhat hindered, especially upon the interaction with CS at such low concentrations. This is in agreement with our observations of only a moderate decrease (<2 mN m^−1^) in the surface pressure during the interaction (ESI,† Fig. S1), and also with previous reports, which showed that the liquid-crystalline phase of the DPPC monolayer amounts to only a small change in the surface area per lipid tail.^3^

**Fig. 4:**
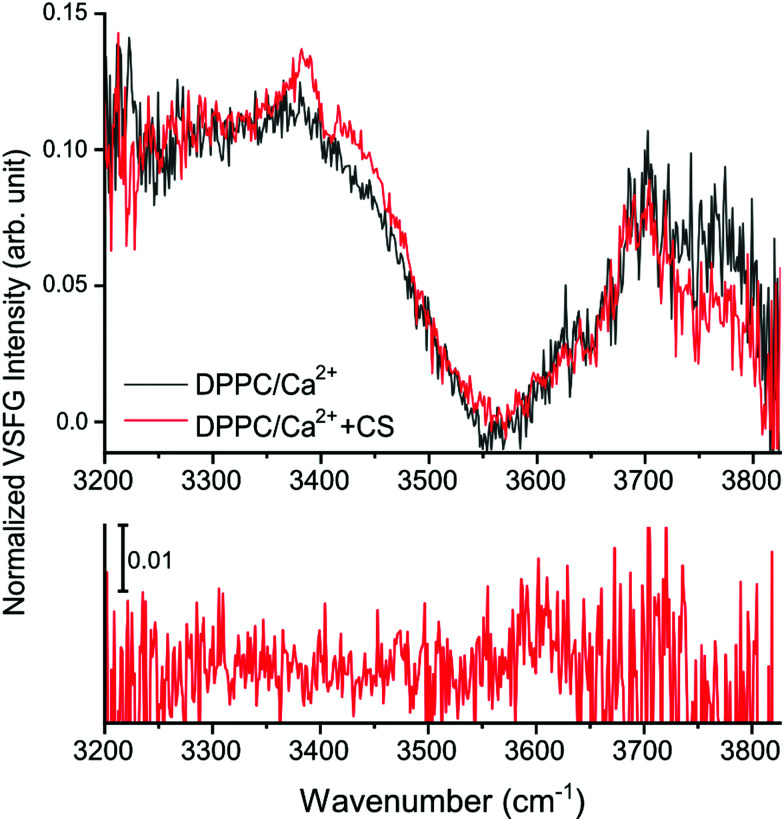
The O–H stretching region of the DPPC spectra with CS (red trace) and without CS (black trace) in *ssp* polarization combination (top), and the VSFG spectrum of the DPPC/Ca^2+^ system with CS in a chiral, *spp* polarization combination (bottom).

In the O–H stretching region, here shown between 3200–3825 cm^−1^ (Fig. 2 and 4 top panel, and ESI,† Fig. S3), the increased hydration of the phosphate group in the presence of CS is confirmed by the increased overall VSFG signal. In addition, new features are observed. As a reference, the VSFG spectra of pure H_2_O and of DPPC without Ca^2+^ were measured to determine the origin of certain spectral features (ESI,† Fig. S3). The broad bands centered at ∼3200 cm^−1^ and ∼3400 cm^−1^ correspond to the ice-like (where the distance between the donated H and the acceptor O atoms, O–H⋯O, is the shortest) and liquid-like (the O–H⋯O distances are somewhat longer) hydrogen bonded O–H stretching vibrations,^2,27,29,30^ respectively. In the presence of DPPC, the overall VSFG intensity increased significantly due to the high DC electric field induced by the interfacial charge density, and thus a third-order contribution to the second order molecular polarization, indicating a well-organized arrangement of water molecules between the choline and phosphate moieties of the lipid headgroups.^4,30^ After the addition of only 2.8 mM CaCl_2_ (making this the first VSFG study at such a low, physiologically relevant Ca^2+^ concentration), the overall intensity of the 3200–3400 cm^−1^ region drastically decreases due to charge neutralization in the zwitterionic headgroups, which causes reorientation and disorder in the interfacial water structure (ESI,† Fig. S3).^2^ In the presence of CS, a large peak appears at 3385 cm^−1^ assigned to the stretching of CS hydroxyl groups interacting with water molecules *via* hydrogen bonding (Fig. 4 and ESI,† Fig. S3).

A negative band at 3408 cm^−1^ is also apparent, probably as a result of destructive interference between the vibrational modes of the liquid-like O–H stretching of the interfacial water molecules (near the choline moiety)^4,30^ and the O–H groups of CS.^31^

Additionally, based on the difference spectra (ESI,† Fig. S4), further new bands appear at 3345, 3430, and ∼3470 cm^−1^. The broad shoulder at ∼3430 cm^−1^ observed in the presence of CS originates from the ordered hydroxyl groups;^31^ the frequency shift towards higher wavenumbers indicates that weaker hydrogen bonds form at the interface when CS is present. The appearance of new bands assigned to CS suggests that CS molecules organize into an ordered layer beneath the DPPC monolayer, as the selection rules of VSFG prevent signal collection from disordered layers or from the bulk. Interestingly, even though the addition of the CS solution caused a decrease in surface pressure (see ESI,† Fig. S1), the intensity in the free O–H stretching region (originating from non-H-bonded water molecules) above 3700 cm^−1^ decreased, contrary to the expected higher contribution of free O–H stretching vibrations. This suggests that the presence of CS, a molecule that is rich in hydroxyl groups and negatively charged sulfates and carboxylates, promotes the binding and thus the stabilization of the water molecules in the lipid monolayer. In the spectrum recorded at a chiral, *spp* polarization combination (Fig. 4 bottom), a weak, broad peak can be observed at ∼3600 cm^−1^ assigned to weakly interacting hydroxyl group vibrations.^32^ Furthermore, bands are visible at ∼3550 cm^−1^ and at ∼3470 cm^−1^ both in achiral and chiral polarization (Fig. 4 and ESI,† Fig. S5), but the low signal-to-noise ratio renders their presence ambiguous. As one or more spectral bands show in the spectrum recorded in *spp* polarization combination (Fig. 3 and ESI,† Fig. S5), we conclude that CS in solution possesses a chiral secondary structure; although, as the intensity of these bands is only a fraction of those originating from CS in the same range recorded in *ssp* polarization combination, it can also be concluded that such a defined secondary structure is present but is not exclusive to the whole molecule. Due to the alternating β1–3 and β1–4 bonds between β-d-glucuronic acid and β-d-*N*-acetylgalactosamine and the ^4^C_1_ conformation of the monosaccharide units, a helical coil secondary structure is most probable. The helical structure of CS has been shown by X-ray crystallography.^33^ In solution, however, this higher order structure has only been postulated^34^ but not confirmed yet.

## Conclusions

In summary, we have successfully studied the interaction of CS with a DPPC monolayer in the presence of Ca^2+^ ions. We found that the interaction causes the reorientation of the lipid headgroups, while the organization of the lipid tails remains almost unchanged. The presence of hydroxyl group vibrations assigned to the CS molecules indicate an organized CS layer beneath the lipid monolayer. Furthermore, to the best of our knowledge, this study provides the first direct proof that CS possesses a linearly chiral secondary structure in solution at a charged biological interface, organized most probably into a helical coil. These results suggest that VSFG spectroscopy is a promising label-free method to explore structural and orientational information at complex, heterogeneous biological (model) interfaces *in situ* in real time and at nanomolar concentrations.

## Conflicts of interest

There are no conflicts to declare. Open Access funding provided by Humboldt-Universität zu Berlin.

## Supplementary Material

CP-023-D1CP01975A-s001
