# Supplementary material for: The interaction of chondroitin sulfate with a lipid monolayer observed by using nonlinear vibrational spectroscopy
Source: Phys Chem Chem Phys. 2021 Jun 7;23(23):13389–95. doi: 10.1039/d1cp01975a (PMC8207512; doi:10.1039/d1cp01975a)
Supplement: CP-023-D1CP01975A-s001 [file CP-023-D1CP01975A-s001.pdf]

Electronic Supplementary Material (ESI) for Physical Chemistry Chemical Physics.
This journal is © the Owner Societies 2021

Electronic Supplementary Information

The interaction of chondroitin sulfate with a DPPC monolayer observed by using nonlinear vibrational spectroscopy

Gergo Peter Szekeres,[a,b] Szilvia Krekic,[c,d,e] Rebecca L. Miller,[f] Mark Mero,[g] Kevin Pagel,*[a,b] Zsuzsanna Heiner*[c]

[a] Institut für Chemie und Biochemie, Freie Universität Berlin, Takustraße 3, 14195 Berlin, Germany

[b] Department of Molecular Physics, Fritz-Haber-Institut der Max-Planck-Gesellschaft, Faradayweg 4-6, 14195 Berlin, Germany

[c] School of Analytical Sciences Adlershof, Humboldt-Universität zu Berlin, Albert-Einstein-Straße 5-11, 12489 Berlin, Germany

[d] Institute of Biophysics, Biological Research Centre, Temesvári krt. 62, 6726, Szeged, Hungary

[e] Doctoral School of Multidisciplinary Medical Sciences, University of Szeged, Dugonics tér 13, 6720, Szeged, Hungary

[f] Copenhagen Center for Glycomics, Department of Cellular and Molecular Medicine, Faculty Sciences, University of Copenhagen, Blegdamsvej 3, DK-2200 Copenhagen N, Denmark

[g] Max Born Institute for Nonlinear Optics and Short Pulse Spectroscopy, Max-Born-Straße 2a, 12489 Berlin, Germany

* Correspondence: kevin.pagel@fu-berlin.de, heinerzs@hu-berlin.de

Materials

In all experiments, Milli-Q water (18.2 MΩ cm) was used, and it is further referred to as “water”. Chloroform (spectroscopic grade, > 99%), methanol (spectroscopic grade, > 99.9%), anhydrous $CaCl_2$, and chondroitin sulfate from shark cartilage (CS) were purchased from Sigma. Dipalmitoyl phosphatidylcholine (DPPC) was purchased from Avanti. Every chemical was used without further purification.

Methods

Surface pressure measurements

The samples were prepared identical to as described in the main text. After the DPPC monolayer stabilized and the organic solvents completely evaporated (10-15 min), 20 µL of a 2.6 mg/mL aqueous CS solution was carefully pipetted into the petri dish while the piston of the Langmuir-Blodgett instrument was kept in the same position. The ~3.5 mN/m decrease in surface pressure upon the addition of 20 µL liquid does not cause observable reorganization in the continuous lipid monolayer in the liquid-crystalline phase,[1,2] as was also found in the VSFG spectra in the C-H stretching region (Figure 2 in the manuscript). Figure S1 summarizes the surface pressure measurements over time upon addition of Ca^{2+} and CS.

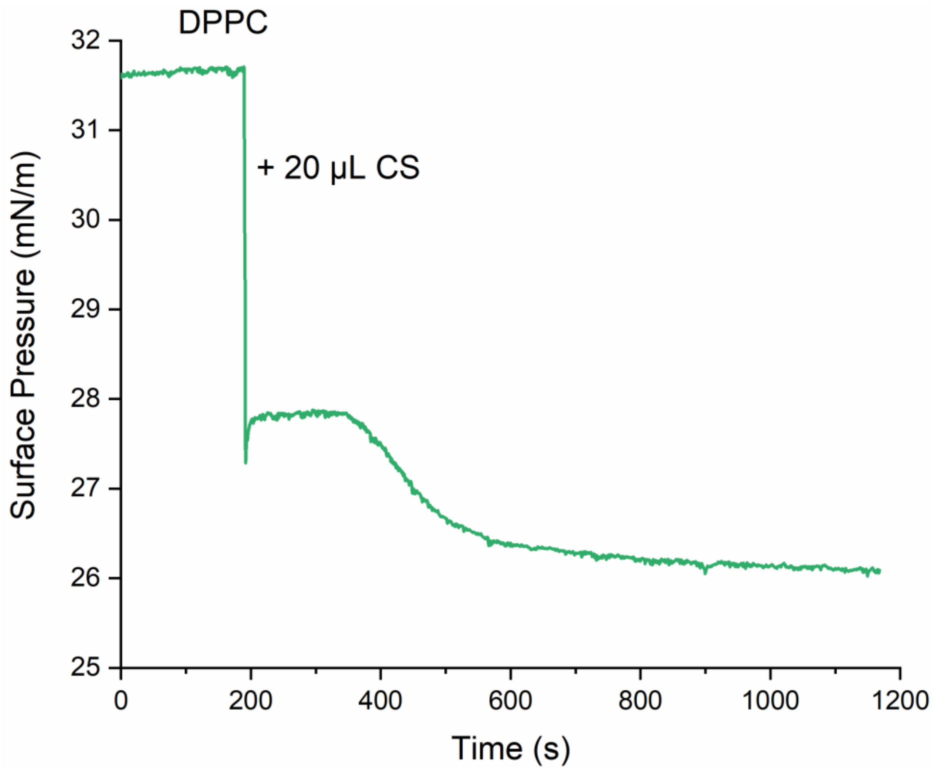

Figure S1. Surface pressure measurements during DPPC-Ca^{2+}-CS interactions.

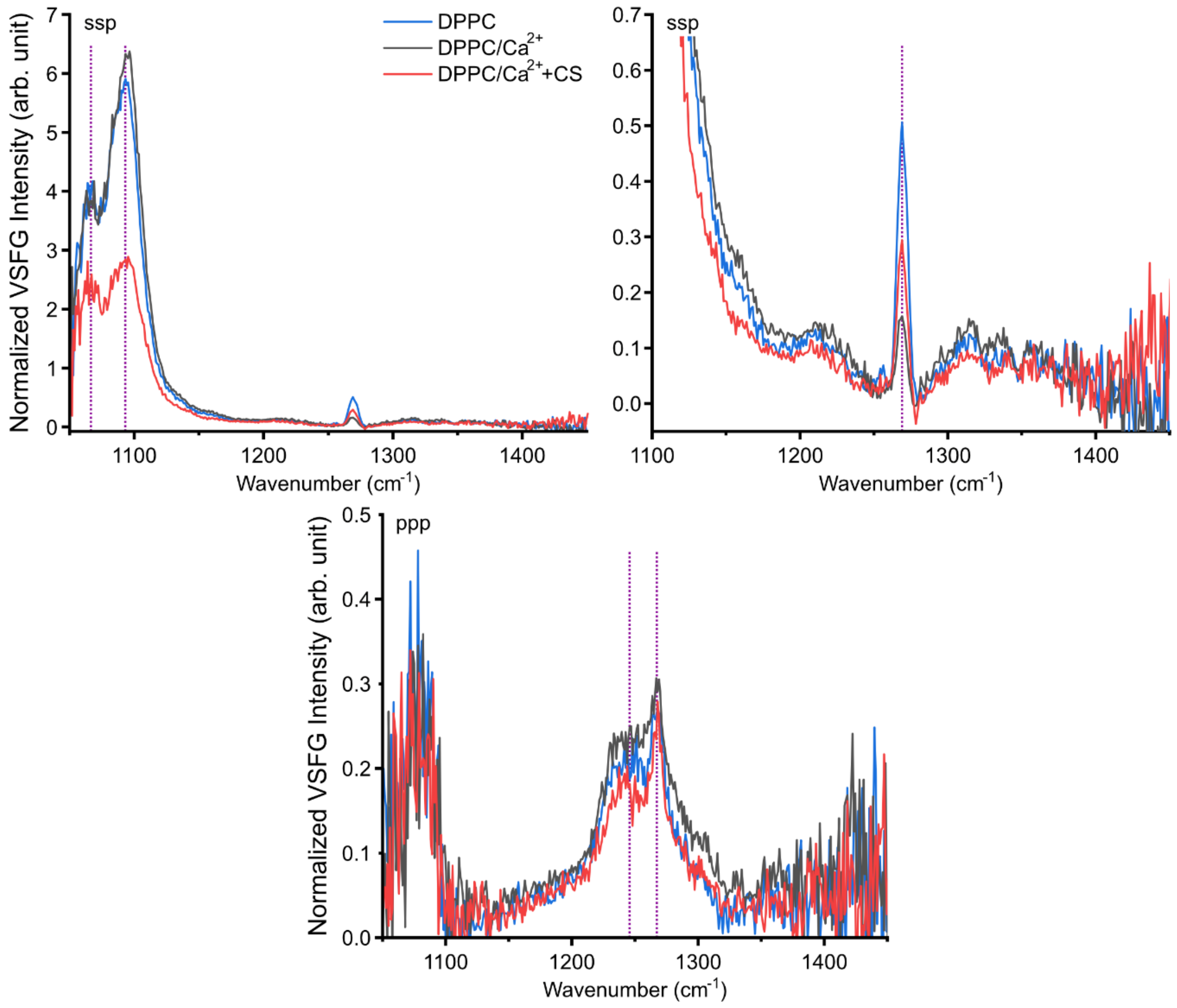

Figure S2. VSFG spectra recorded in the fingerprint spectral region of a DPPC monolayer with pure water (blue trace), 2.8 mM Ca^{2+} (black trace), and 2.8 mM Ca^{2+} with CS as sub-phase (red trace) both in *ssp* polarization combination (top left, with inset on the right) and in *ppp* polarization combination (bottom).

Lorentzian peak fitting

Lorentzian peaks were fitted to the VSFG spectra based on equation (1) as also described by Yesudas et al.[3]

$$VSFG\ intensity\ (\omega) = \left| P_{NR} e^{i\Phi} + \sum_{i=1}^{\nu} \frac{Q_v}{\omega - \omega_\nu - i\Gamma_v} \right|^2 , \qquad (1)$$

Where Q_v, ω_v, *and* Γ_v are the strength, frequency and damping factor of the vth Lorentzian peak. The first element of the sum accounts for the non-resonant contribution with amplitude P_{NR} and phase Φ. The Lorentzian peaks were fitted to the normalized VSFG spectra with a home-written script based on equation (1) in the software Igor Pro 8, which allowed for describing both constructive- and destructive interferences. The spectra of the samples without CS were fitted with 7 Lorentzian peaks and the non-resonant contribution, while the spectrum of the sample with CS was fitted with an additional peak as well. Two further peaks were included in the fitting at higher wavenumbers to ensure proper convergence, which peaks are visibly present at ~1188 and 1202 cm^{-1}, but due to their near-zero intensity, their fitting could only be done with large errors, and therefore they were not included in Table S1 presenting quantitative spectral information.

Table S1. Parameters extracted from the Lorentzian peak fitting of the VSFG spectra of DPPC with subphases of H_2O, 2.8 mM Ca^{2+}, and 2.8 mM Ca^{2+} with <200 nM CS in the 1050-1200 cm^{-1} spectral region.

Sample	ω_v	Q_v	Γ_v
DPPC	1066.81 ± 0.19	10.69 ± 0.79	9.18 ± 0.23
	1074.90 ± 0.22	0.93 ± 0.36	4.10 ± 0.71
	1083.79 ± 0.41	9.61 ± 2.89	8.27 ± 0.92
	1092.54 ± 0.45	8.59 ± 5.41	7.91 ± 1.69
	1098.79 ± 1.05	6.81 ± 3.56	8.72 ± 0.77
	1132.12 ± 1.10	0.33 ± 0.18	5.18 ± 2.28
	1155.46 ± 2.30	0.75 ± 0.56	11.65 ± 5.82
DPPC/Ca^{2+}	1065.94 ± 0.15	9.21 ± 0.45	8.62 ± 0.06
	1074.92 ± 0.17	2.20 ± 0.22	5.94 ± 0.20
	1083.76 ± 0.25	9.28 ± 1.86	8.30 ± 0.58
	1093.11 ± 0.25	11.24 ± 3.64	8.29 ± 0.94
	1099.96 ± 0.55	5.76 ± 2.16	8.00 ± 0.71
	1133.59 ± 0.77	0.25 ± 0.12	4.20 ± 1.58
	1154.52 ± 1.41	0.58 ± 0.25	7.58 ± 2.27
DPPC/Ca^{2+} + CS	1067.48 ± 0.49	17.60 ± 1.31	13.09 ± 0.02
	1074.59 ± 1.63	5.87 ± 0.82	11.38 ± 0.04
	1083.51 ± 0.41	7.15 ± 2.58	7.47 ± 0.96
	1090.44 ± 0.28	4.24 ± 2.43	6.19 ± 1.35
	1096.14 ± 0.41	2.41 ± 1.27	5.44 ± 0.82
	1111.68 ± 0.65	-3.03 ± 1.09	10.45 ± 1.43
	1136.89 ± 1.75	0.18 ± 0.07	6.22 ± 2.07
	1155.17 ± 1.38	0.94 ± 0.22	8.74 ± 1.41

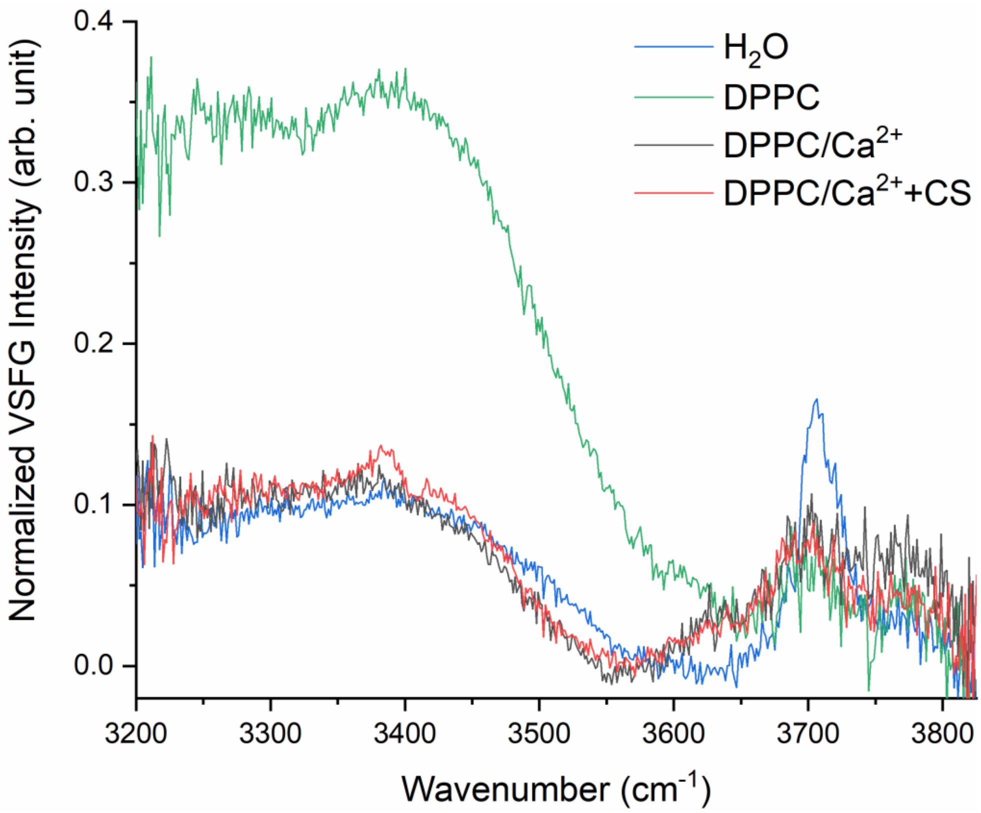

Figure S3. VSFG spectra of pure H_2O, DPPC, DPPC with 2.8 mM Ca^{2+}, and DPPC with 2.8 mM Ca^{2+} and CS at the air-liquid interface obtained at *ssp* polarization combination.

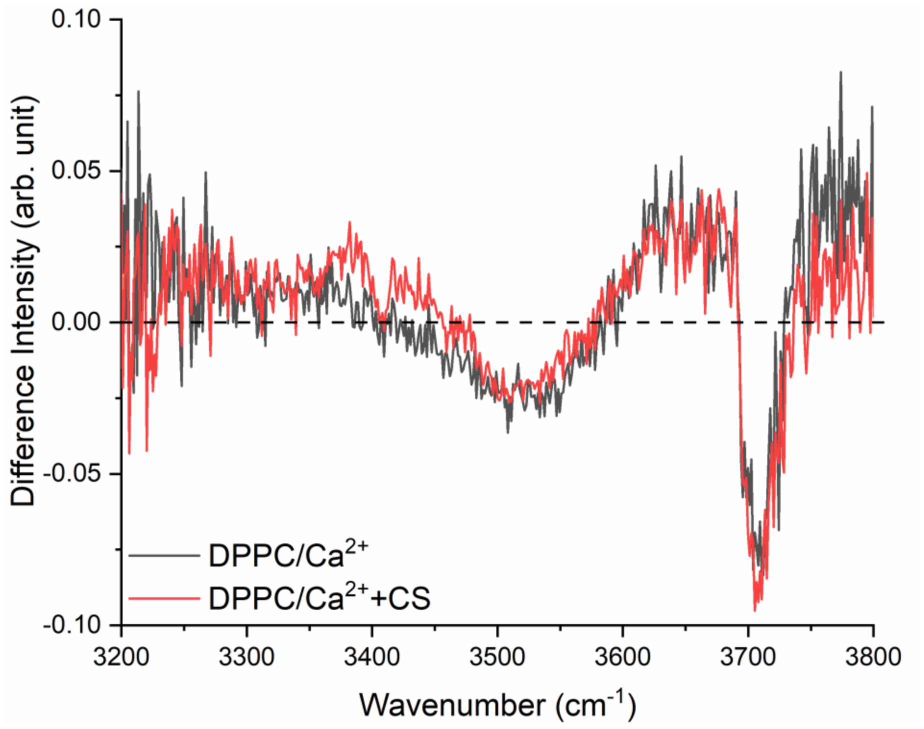

Figure S4. Difference *ssp* VSFG spectra of the samples with DPPC with 2.8 mM Ca^{2+} and DPPC with 2.8 mM Ca^{2+} and CS created by subtracting the VSFG spectrum of pure H_2O from the spectrum of the respective sample.

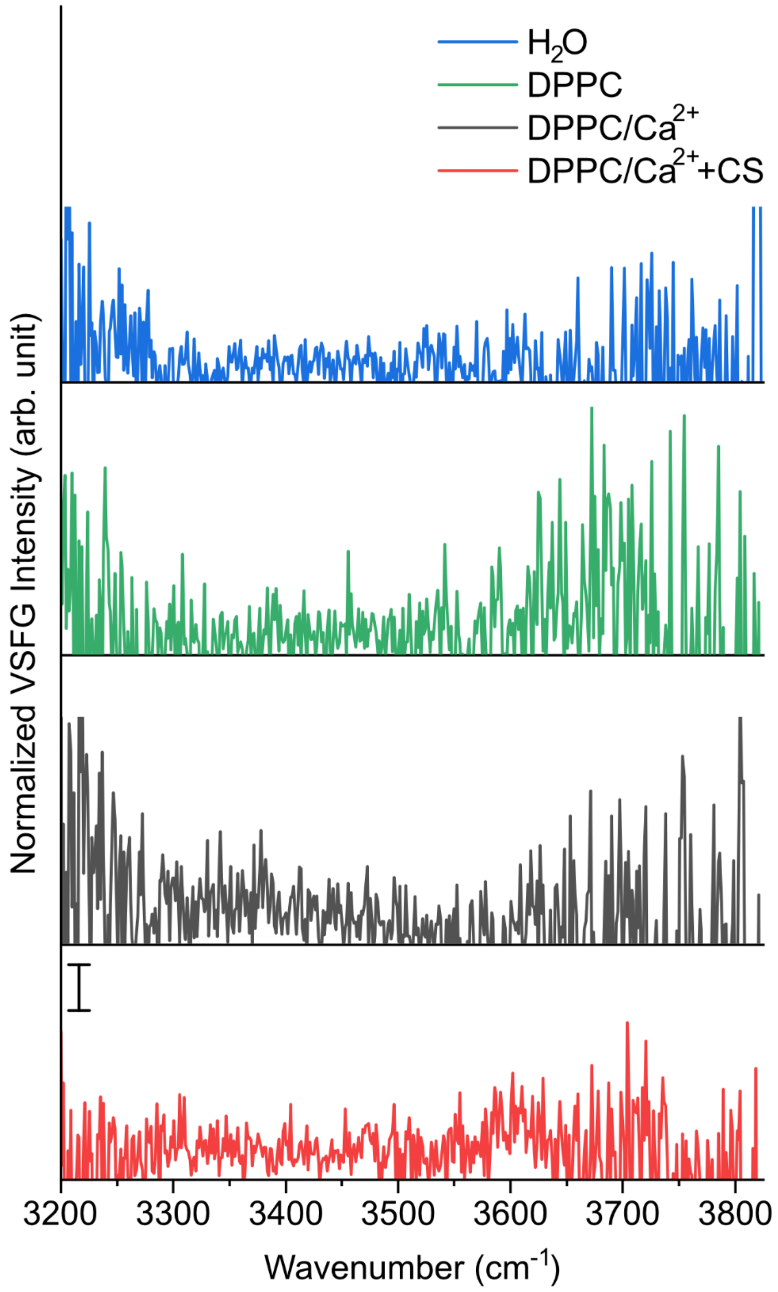

Figure S5. VSFG spectra of pure H_2O, DPPC, DPPC with 2.8 mM Ca^{2+}, and DPPC with 2.8 mM Ca^{2+} and CS in spp polarization combination. The scale bar corresponds to 0.01.

References

1 N. N. Casillas-Ituarte, X. Chen, H. Castada and H. C. Allen, *J. Phys. Chem. B*, 2010, **114**, 9485–9495.

2 G. A. Lawrie, I. R. Gentle and G. T. Barnes, *Colloids Surfaces A Physicochem. Eng. Asp.*, 2000, **171**, 217–224.

3 F. Yesudas, M. Mero, J. Kneipp and Z. Heiner, *Anal. Bioanal. Chem.*, 2019, **411**, 4861–4871.
